# The effect of herbal feed additive on the growth performance, carcass characteristics and meat quality of broiler chickens fed low-energy diets

**DOI:** 10.5194/aab-62-33-2019

**Published:** 2019-01-22

**Authors:** Krzysztof Lipiński, Zofia Antoszkiewicz, Sylwia Kotlarczyk, Magdalena Mazur-Kuśnirek, Joanna Kaliniewicz, Zbigniew Makowski

**Affiliations:** Department of Animal Nutrition and Feed Science, University of Warmia and Mazury in Olsztyn, 10-719 Olsztyn, Poland

## Abstract

The aim of this study was to determine the effect of herbal feed additive on growth performance, carcass characteristics, the chemical composition of breast muscles and selected meat quality parameters in broiler chickens. The experiment was performed on 1080 Ross 308 chickens, which were randomly divided into six groups with six replicates per group. In experimental groups, dietary energy concentration was reduced by 0.10 or 0.25 MJ kg${}^{-1}$, and the diets were or were not supplemented with the Superliv herbal formula at 500 g t${}^{-1}$ of diet. A statistical analysis revealed that Superliv had a beneficial influence on the final body weights (BWs) of birds, average daily gain (ADG), feed conversion ratio (FCR), European Efficiency Index (EEI) and the proportion of heart relative to total carcass weight. However, meat acidity measured 15 min post mortem was higher in experimental groups. A decrease in dietary energy concentration contributed to a highly significant increase in the water-holding capacity (WHC) of meat and natural drip loss, an increase in pH${}_{15}$, an increase in color lightness, a decrease in redness, an increase in the fat content of meat, and a decrease in malondialdehyde (MDA) concentration.

**Table 1:** Composition and nutritional value of diets for broiler chickens.

Composition	Starter I	Grower I	Grower II	Finisher
	days 0–10	days 11–21	days 22–35	days 36–42
Soybean meal	29.66	23.89	20.84	16.97
Wheat	29.34	36.17	41.39	45.59
Maize	25.00	20.00	15.00	10.00
Triticale	5.00	6.00	8.00	10.00
Rapeseed meal	4.00	6.00	6.00	8.00
Soybean oil	3.10	4.30	5.30	6.31
DL methionine	0.25	0.22	0.23	0.18
L lysine	0.24	0.29	0.32	0.35
L threonine	0.04	0.08	0.09	0.09
Limestone	1.22	1.20	1.17	1.10
Monocalcium phosphate	1.12	0.88	0.70	0.54
Sodium bicarbonate	0.21	0.16	0.14	0.12
Premix	0.50	0.50	0.50	0.50
Nutritional value metabolizable energy, MJ kg${}^{-1}$	12.38	12.80	13.18	13.47
Crude protein, g kg${}^{-1}$	215.0	200.0	190.0	180.0
Lysine, g kg${}^{-1}$	12.80	12.00	11.50	11.00
Methionine + cystine, g kg${}^{-1}$	9.50	9.00	8.70	8.10
Calcium, g kg${}^{-1}$	9.50	9.00	8.50	8.00
Available phosphorus g kg${}^{-1}$	4.50	4.00	3.60	3.30
Sodium, g kg${}^{-1}$	1.80	1.60	1.60	1.60

**Table 2:** Experimental design.

Group		Experimental factor
1	Positive control (PC)	–
2	PC + herbal feed additive	+ herbal feed additive 500 g t${}^{-1}$
3	Negative control (NC)	-0.10 MJ kg${}^{-1}$
4	NC + herbal feed additive	-0.10 MJ kg${}^{-1}$ + herbal feed additive 500 g t${}^{-1}$
5	Negative control (NC)	-0.25 MJ kg${}^{-1}$
6	NC + herbal feed additive	-0.25 MJ kg${}^{-1}$ + herbal feed additive 500 g t${}^{-1}$

## 1 Introduction

Stimulation of the immune system and antioxidant defense in poultry has been a topical issue since the introduction of the ban on the use of antibiotics as growth promoters in animal feed. There is a demand for alternative feed additives that promote the growth of animals without negative side effects such as the antibiotic resistance of pathogenic strains. The beneficial influence of probiotics, prebiotics, acidifiers, enzymes and herbs on gut microbiota in monogastric animals has been widely researched. Herbs and spices contain numerous active ingredients which can exert various effects (bactericidal, immunomodulatory and antioxidant) on animals. Thus, they can affect the health status and productivity of animals as well as the quality of animal products (Madhupriya et al., 2018). However, the intensity and direction of the effects exerted by herbal preparations are difficult to predict. They are mostly determined by the concentrations of active ingredients but may also be influenced by the presence of auxiliary substances and interactions with other active ingredients or feed components (Hashemi and Davoodi, 2011; Ogbuewu et al., 2018). Herbs and herbal extracts have been used in poultry production for years. Superliv is one of such herbal formulations. Due to the presence of multiple active ingredients, Superliv may exert multidirectional effects on animals, including the stimulation of their growth, performance and digestion. It may also act as a hepatoprotective agent that neutralizes toxins. The composition of diets supplemented with herbal extracts can be modified, e.g., by reducing their energy concentration. Stimulation of the liver's functions may affect a superior nutrient's digestibility and have a gainful effect on energy utilization. Thus, it can allow us to achieve high growth performance with lower energy concentrations in the diets.

The objective of this study was to determine the effect of the Superliv herbal formula on the growth performance, carcass characteristics and meat quality of broiler chickens fed low-energy diets.

**Table 3:** Results of growth trial.

Experimental factor	Final body	Average daily	Feed conversion	Mortality	EEI${}^{*}$,
	weight, kg	gain, g	ratio, kg kg${}^{-1}$	rate, %	points
K	2.81${}^{\text{BCc}}$	65.9${}^{\text{BCc}}$	1.62${}^{\text{BCc}}$	3.33	398.0${}^{\text{By}}$
K+S	2.85${}^{\text{bcx}}$	67.1${}^{\text{y}}$	1.54${}^{\text{Aa}}$	0.00	440.5${}^{\text{Aa}}$
K-0.10 MJ kg${}^{-1}$	2.85${}^{\text{bcx}}$	66.9${}^{\text{bcxy}}$	1.62${}^{\text{ABCby}}$	4.67	400.5${}^{\text{byz}}$
K-0.10 MJ kg${}^{-1}+S$	2.93${}^{\text{Aa}}$	68.8${}^{\text{Aax}}$	1.57${}^{\text{ABabx}}$	4.00	426.7${}^{\text{abxy}}$
K-0.25 MJ kg${}^{-1}$	2.78${}^{\text{Cy}}$	65.3${}^{\text{Cz}}$	1.64${}^{\text{C}}$	4.00	368.6${}^{\text{Bc}}$
K-0.25 MJ kg${}^{-1}+S$	2.90${}^{\text{ABab}}$	68.1${}^{\text{ABab}}$	1.57${}^{\text{Babx}}$	4.29	423.5${}^{\text{bax}}$
Analysis of variance					
SEM	0.013	0.313	0.009	0.53	5.006
P	0.003	0.003	0.002	0.12	0.004
Energy					
K	2.83${}^{\text{x}}$	66.46	1.58	1.67${}^{\text{x}}$	419.25
K-0.10 MJ kg${}^{-1}$	2.88${}^{\text{y}}$	67.69	1.6	4.33${}^{\text{y}}$	413.62
K-0.25 MJ kg${}^{-1}$	2.84	66.68	1.61	4.00${}^{\text{y}}$	405
Superliv					
-	2.81${}^{\text{A}}$	66.09${}^{\text{A}}$	1.63${}^{\text{A}}$	4.00	393.73${}^{\text{A}}$
+	2.89${}^{\text{B}}$	67.98${}^{\text{B}}$	1.56${}^{\text{B}}$	2.67	430.22${}^{\text{B}}$
Analysis of variance	P				
Energy (E)	0.099	0.112	0.427	0.073	0.347
Superliv (S)	<0.001	<0.001	<0.001	0.187	<0.001
Interaction (E×S)	0.388	0.396	0.663	0.357	0.812
SEM	0.013	0.313	0.009	0.53	0.313

${}^{*}$ European Efficiency Index. ${}^{\text{a, b}}\;P\leq 0.05$. ${}^{\text{A, B, C}}\;P\leq 0.01$. ${}^{\text{x/y/z}}\;0.05<P\leq 0.10$.

**Table 4:** Carcass characteristics.

		Percentage in total carcass weight, %			
Specification	Dressing	Breast	Abdominal	Heart	Liver
	percentage, %	muscles	fat		
Diet					
K	73.11${}^{\text{y}}$	30.97	1.03	0.64${}^{\text{yz}}$	2.99${}^{\text{x}}$
K+S	73.56	30.55	0.88	0.70${}^{\text{ax}}$	2.70
K-0.10 MJ kg${}^{-1}$	73.79	30.90	0.94	0.62${}^{\text{bc}}$	3.00${}^{\text{x}}$
K-0.10 MJ kg${}^{-1}+S$	73.43	30.58	0.89	0.64${}^{\text{y}}$	2.76
K-0.25 MJ kg${}^{-1}$	73.63	30.42	1.09	0.58${}^{\text{cz}}$	2.69
K-0.25 MJ kg${}^{-1}+S$	74.41${}^{\text{x}}$	30.48	0.98	0.66${}^{\text{ab}}$	2.64${}^{\text{y}}$
Analysis of variance					
SEM	0.178	0.207	0.040	0.010	0.054
P	0.428	0.965	0.652	0.013	0.206
Energy					
K	73.34	30.76	0.96	0.67${}^{\text{x}}$	2.85
K-0.10 MJ kg${}^{-1}$	73.61	30.74	0.92	0.63${}^{\text{y}}$	2.88
K-0.25 MJ kg${}^{-1}$	74.02	30.45	1.03	0.62${}^{\text{y}}$	2.66
Superliv					
-	73.51	30.76	1.02	0.61${}^{\text{A}}$	2.89${}^{\text{x}}$
+	73.80	30.53	0.92	0.67${}^{\text{B}}$	2.70${}^{\text{y}}$
Analysis of variance	P				
Energy (E)	0.297	0.807	0.502	0.076	0.206
Superliv (S)	0.416	0.596	0.198	0.004	0.074
Interaction (E×S)	0.405	0.891	0.894	0.461	0.641
SEM	0.178	0.207	0.040	0.010	0.054

${}^{\text{a, b}}$ P≤0.05. ${}^{\text{A, B, C}}$ P≤0.01. ${}^{\text{x/y/z}}$ 0.05<P≤0.10.

## 2 Materials and methods

The experiment was approved by the Local Ethics Committee for Animal Experimentation in Olsztyn, Poland. The experiment was conducted on 1080 Ross 308 broiler chickens (male), which were randomly divided into six groups with six replicates per group and 30 birds per subgroup. Broiler chickens were raised on litter under standard housing conditions. The experiment lasted for 6 weeks. The birds were fed complete diets, and the feeding period was divided into four phases. The composition and nutritional value of diets were adjusted to meet the changing nutrient requirements of birds (Table 1). Control group birds received diets with standard energy concentration and without the herbal formula. In experimental groups, dietary energy concentration was reduced by 0.10 or 0.25 MJ kg${}^{-1}$, and the diets were or were not supplemented with the herbal formula (Table 2). The Superliv herbal formula (Ayurvet; Delhi, India) contains the following herbs: *Ichnocarpus frutescens*, *Terminalia chebula*, *Sida cordifolia*, *Terminalia arjuna*, *Phyllanthus emblica*, *Tephrosia purpurea*, *Fumaria indica*, *Andrographis paniculata*, *Azadirachta indica*, *Tinospora cordifolia*, *Achyranthes aspera*, *Boerhavia diffusa*, *Solanum nigrum*, *Citrullus colocynthis*, *Eclipta alba*, *Aphanamixis polystachya* and *Phyllanthus niruri*. The growth-promoting and liver protective effects of the active ingredients contained in the above herbs have been documented. Superliv has been found to improve nutrient utilization and feed efficiency.

**Table 5:** Chemical composition of breast muscles.

Specification	Dry	Crude	Crude	Crude	Malondialdehyde,
	matter, %	ash, %	protein, %	fat, %	mg kg${}^{-1}$
Diet					
K	26.36	1.15	23.00	0.91${}^{\text{B}}$	0.63${}^{\text{abxy}}$
K+S	26.11	1.16	22.76	1.27${}^{\text{bcyz}}$	0.67${}^{\text{axy}}$
K-0.10 MJ kg${}^{-1}$	26.58	1.12	22.46	1.64${}^{\text{Axy}}$	0.58${}^{\text{abyz}}$
K-0.10 MJ kg${}^{-1}+S$	25.97	1.16	22.51	1.21${}^{\text{cz}}$	0.38${}^{\text{zv}}$
K-0.25 MJ kg${}^{-1}$	26.22	1.16	22.57	1.74${}^{\text{Aabx}}$	0.34${}^{\text{bcv}}$
K-0.25 MJ kg${}^{-1}+S$	26.58	1.16	22.76	1.84${}^{\text{Aa}}$	0.27${}^{\text{c}}$
Analysis of variance					
SEM	0.121	0.008	0.110	0.085	0.045
P	0.647	0.656	0.756	0.002	0.021
Energy					
K	26.24	1.16	22.88	1.09${}^{\text{Aa}}$	0.65${}^{\text{Ax}}$
K-0.10 MJ kg${}^{-1}$	26.28	1.14	22.48	1.43${}^{\text{b}}$	0.48${}^{\text{y}}$
K-0.25 MJ kg${}^{-1}$	26.40	1.16	22.66	1.79${}^{\text{Ba}}$	0.31${}^{\text{Bx}}$
Superliv					
-	26.39	1.14	22.68	1.43	0.52
+	26.22	1.16	22.68	1.44	0.44
Analysis of variance			P		
Energy (E)	0.861	0.538	0.378	<0.001	0.005
Superliv (S)	0.506	0.275	0.998	0.931	0.317
Interaction (E×S)	0.289	0.679	0.748	0.056	0.419
SEM	0.121	0.008	0.110	0.085	0.045

${}^{\text{a, b, c}}$ P≤0.05. ${}^{\text{A, B, C}}$ P≤0.01. ${}^{\text{x/y/z}}$ 0.05<P≤0.10.

The measurements performed during the experiment were used to calculate the feed conversion ratio (FCR) and European Efficiency Index (EEI). After slaughter, two birds per subgroup were used to determine carcass characteristics (dressing percentage, percentage content of breast muscles, abdominal fat, heart and liver in total carcass weight), the proximate chemical composition of breast muscles (content of dry matter, crude ash, crude protein and crude fat) according to AOAC (2005), the physicochemical parameters of breast muscles, pH${}_{15}$, pH${}_{24}$, color in CIE L*a*b* (L* – lightness; a* – redness; b* – yellowness) (CIE, 1978) with the Hunter Lab Mini Scan XE Plus spectrophotometer, 24 h post mortem, water-holding capacity (WHC; by the Grau and Hamm method, 1952, modified by Pohja and Niinivaara, 1957), natural drip loss (by the method proposed by Honikel, 1998) and malondialdehyde (MDA) concentration (as described by Sorensen and Jorgensen, 1996). The results were processed by two-way analysis of variance (ANOVA) and Duncan's test. Arithmetic means (x), standard error of the mean (SEM), significance level (P≤0.05, P≤0.01) and statistical tendencies x, y (P>0.05, P≤0.1) were calculated using STATISTICA 12.0 software.

## 3 Results

The Superliv herbal formula had a positive effect on nearly all performance parameters in broiler chickens. The product's beneficial influence on mortality rates was not statistically significant, whereas mortality tended to increase with decreasing dietary energy concentration. Low-energy diets had no significant effect on the remaining parameters of growth performance. No interactions were found between the experimental factors (Table 3).

Superliv neither reduced dietary energy concentration nor affected carcass dressing percentage or the percentage content of breast muscles and abdominal fat in the carcass (Table 4). Highly significant differences were noted in the percentage content of heart in total carcass weight (Table 4). Heart weight increased in chickens fed diets supplemented with Superliv and tended to decrease with decreasing dietary energy concentration.

**Table 6:** Meat quality parameters.

					Color, CIE Lab		
Specification	Water-holding	Natural drip	pH${}_{15}$	pH${}_{24}$	L	a	b
	capacity, cm${}^{2}$	loss, %					
Diet							
K	1.67${}^{\text{A}}$	4.17${}^{\text{A}}$	6.36${}^{\text{ABa}}$	6.08	61.19${}^{\text{Aax}}$	6.36${}^{\text{Aa}}$	14.39
K+S	2.25${}^{\text{ABa}}$	4.50${}^{\text{A}}$	6.41${}^{\text{ab}}$	5.98	62.82${}^{\text{ab}}$	6.30${}^{\text{Aa}}$	14.01
K-0.10 MJ kg${}^{-1}$	3.12${}^{\text{BCbx}}$	7.22${}^{\text{B}}$	6.33${}^{\text{Aa}}$	5.96	63.04${}^{\text{aby}}$	6.23${}^{\text{Aabx}}$	14.51
K-0.10 MJ kg${}^{-1}+S$	3.22${}^{\text{BCb}}$	7.52${}^{\text{B}}$	6.48${}^{\text{BCb}}$	6.02	63.31${}^{\text{aby}}$	5.44${}^{\text{bcy}}$	14.38
K-0.25 MJ kg${}^{-1}$	3.97${}^{\text{Cy}}$	7.31${}^{\text{B}}$	6.53${}^{\text{Cbc}}$	6.04	65.56${}^{\text{Bc}}$	4.47${}^{\text{Bd}}$	14.20
K-0.25 MJ kg${}^{-1}+S$	3.57${}^{\text{C}}$	7.62${}^{\text{B}}$	6.52${}^{\text{Cbc}}$	6.05	63.99${}^{\text{bc}}$	5.30${}^{\text{c}}$	14.41
Analysis of variance							
SEM	0.155	0.335	0.017	0.015	0.321	0.141	0.167
P	<0.001	<0.001	<0.001	0.190	0.002	<0.001	0.970
Energy							
K	1.97${}^{\text{A}}$	4.33${}^{\text{A}}$	6.38${}^{\text{A}}$	6.03	62.01${}^{\text{Ax}}$	6.33${}^{\text{Ax}}$	14.20
K-0.10 MJ kg${}^{-1}$	3.17${}^{\text{B}}$	7.37${}^{\text{B}}$	6.41${}^{\text{AB}}$	5.99	63.18${}^{\text{ay}}$	5.84${}^{\text{Ay}}$	14.45
K-0.25 MJ kg${}^{-1}$	3.17${}^{\text{B}}$	7.47${}^{\text{B}}$	6.52${}^{\text{B}}$	6.04	64.77${}^{\text{Bb}}$	4.88${}^{\text{B}}$	14.31
Superliv							
-	2.93	6.23	6.41${}^{\text{a}}$	6.03	63.27	5.69	14.37
+	3.01	6.55	6.47${}^{\text{b}}$	6.02	63.37	5.68	14.27
Analysis of variance	P						
Energy (E)	<0.001	<0.001	<0.001	0.320	0.001	<0.001	0.845
Superliv (S)	<0.001	0.587	0.036	0.676	0.853	0.977	0.775
Interaction (E×S)	0.724	0.999	0.073	0.082	0.079	0.020	0.791
SEM	0.300	0.335	0.017	0.015	0.321	0.141	0.167

${}^{\text{a, b, c}}$ P≤0.05. ${}^{\text{A, B, C}}$ P≤0.01. ${}^{\text{x/y/z}}$ 0.05<P≤0.10.

Table 5 presents the chemical composition and MDA content of breast muscles. Neither the chemical composition of breast muscles nor MDA concentration were affected by the herbal feed additive. Fat content and susceptibility to lipid peroxidation were influenced by energy concentration in the ration. Crude fat content was highly significantly and significantly higher in the breast muscles of broilers fed low-energy diets. The breast muscles of birds from groups 5 and 6 had the lowest MDA concentration and the highest fat content. The decrease in dietary energy concentration had a significant effect on lipid peroxidation.

Superliv had no significant influence on the physicochemical properties of meat, except for pH${}_{15}$, which increased slightly in response to the herbal formula. Reduced energy concentration in the ration had a highly significant effect on nearly all meat quality parameters (excluding pH${}_{24}$ and the contribution of yellowness). A considerable decrease was noted in WHC and natural drip loss, and a minor increase was observed in pH${}_{15}$ and color lightness. The contribution of redness decreased in response to reduced dietary energy concentration (Table 6).

## 4 Discussion

Singh et al. (2009), who also tested Superliv in 500 g t${}^{-1}$ of feed, noted higher final body weights and lower FCR values in broiler chickens administered the herbal liver tonic compared with the control group. Similar results were reported by Sharma et al. (2008), who investigated the efficacy of two herbal products – a liver tonic (Superliv DS, 250 g t${}^{-1}$) and a growth promoter (Xlivpro) – in broiler chickens. Both products contributed to an increase in the final body weights of birds and lower feed intake per kilogram of body weight gain. In a field trial where dietary supplementation with Superliv liquid was analyzed under farm conditions, an improvement in growth performance was noted in supplemented broilers, but the increase in average daily gain (ADG) was not statistically significant in the last week of fattening (Bhattacharyya, 2013). According to the above authors, the efficacy of Superliv was higher in birds exposed to stress, pathogens and changes in energy concentration in the ration. Herbs administered to turkeys had no influence on their performance (Lipiński et al., 2011).

Previous research has not confirmed a positive effect of herbal extracts on heart weight in broiler chickens. No significant differences in heart weight were found between control and experimental groups in studies investigating mixtures of herbal plants (Al-Kassie and Witwit, 2010; Amad et al., 2011) or single herbs (Demir et al., 2008; Al-Kassie, 2010). The differences between the above findings and our results could be due to different dietary inclusion levels of herbs and different concentrations of active ingredients in the tested products. Inappropriate feed formulation and other nutritional mistakes may lead to morphological changes in the liver including enlargement, thus negatively affecting broiler productivity. Due to its composition, Superliv was expected to exert hepatoprotective, immunomodulatory, antioxidant and growth-promoting effects (Singh et al., 2009). In the present study, liver weight tended to decrease in response to the herbal feed additive. Our results corroborate the findings of Bhattacharyya et al. (2013), who observed enhanced activity of Superliv liquid under reduced welfare standards, in this case resulting from liver overload due to an imbalance between dietary protein and energy intake. In a study by Sharma et al. (2008), broiler chickens administered Superliv at 250 g t${}^{-1}$ were characterized by a lower liver weight than control group birds.

Due to the high antioxidant potential of active ingredients, numerous studies have investigated the effects of herbs on the oxidative stability of lipids in poultry meat, but their results are inconclusive. Some studies have revealed a decrease in MDA concentration after the application of herbal essential oils. Giannenas et al. (2005) demonstrated that the MDA content of breast muscles was significantly lower in broiler chickens fed diets supplemented with oregano oil, compared with the control group. When alpha-tocopheryl acetate was applied with oregano, MDA concentration decreased steadily. Luna et al. (2010) analyzed pure active ingredients isolated from oregano, thymol and carvacrol. Similarly to our experiment, the herbal feed additive had no significant effect on the oxidative stability of lipids in breast muscles. In the cited study, lipids extracted from thigh muscles were more susceptible to oxidation. Botsoglou et al. (2004) administered a mixture of herbal essential oils to broiler chickens with drinking water. Lipids in breast muscles were significantly less susceptible to oxidation in birds receiving oregano-derived essential oils supplemented through drinking water. Such an effect was also observed by Basmacioglu et al. (2004), who analyzed oregano and rosemary essential oils. In the current study, Superliv did not contribute to a significant decrease in MDA levels.

Acidity is measured 15 min post mortem to distinguish between normal-quality meat and PSE/DFD (pale, soft, exudative/dark, firm, dry) meat. Research shows that the normal pH range of meat is 5.8 (Wójcik et al., 2011) or 5.9 (Żywica et al., 2011) to 6.2 (Wójcik et al., 2011; Żywica et al., 2011). The values below 5.7 and above 6.4 are indicative of PSE and DFD meat, respectively. In the present study, pH${}_{15}$ was 6.36 in the control group, and it ranged from 6.3 to 6.5 in experimental groups. The average pH${}_{24}$ value of chicken meat varies between 5.6 and 6.1 (Berri et al., 2005; Gornowicz and Pietrzak, 2008; Mikulski et al., 2010; Rycielska et al., 2010). The pH${}_{24}$ values determined in our study remain within the upper limit of the normal range. Higher pH levels are associated with a shorter shelf life of meat since they may promote microbial growth, as well as with a darker color and higher WHC. Such relationships, although nonlinear, were also noted in our study, and they were reflected in the differences between groups 5 and 6 vs. the control treatment. The results of meat color analysis, in particular the values of parameter L (lightness), are difficult to interpret. The results of international studies vary with regard to the L values of PSE, DFD and normal-quality meat. The latter were determined to be 43–56 in Poland (Lesiów et al., 2003), 41–66 in Italy (Petracci et al., 2004) and 45–67 in the United Kingdom (Wilkins et al., 2000). According to Branscheid et al. (2004), DFD meat can occur at the normal threshold values (51–57), but normal meat cannot be reliably distinguished from meat with qualitative defects based solely on color lightness. Therefore, advanced methods of meat quality assessment should be applied to identify meat parameters that are the most reliable indicators of quality and to analyze correlations between them. One such technique is cluster analysis (Karpiesiuk et al., 2016).

Water-holding capacity is one of the most important quality attributes of meat, in particular with regard to its processing suitability. In the present study, a decrease in WHC was observed, but it was not associated with the tested herbal preparation. The available literature provides no information on the effect of low-energy diets fed to broiler chickens on meat quality, including WHC and natural drip loss. The results of studies investigating the efficacy of herbal additives in broiler chicken nutrition are inconclusive. Reis et al. (2018) reported an increase in the WHC of meat in broiler chickens receiving a feed additive based on essential oils. In a study by Puvača et al. (2014), the addition of garlic powder to broiler diets also led to a significant increase in WHC. On the other hand, no significant differences in the WHC of meat were found between dietary treatments in broiler chickens fed diets supplemented with lavender essential oil (Küçükyilmaz et al., 2016) and lavender powder (Salajegheh et al., 2018).

## 5 Conclusions

Low-energy diets had a negative effect on broiler chicken productivity, whereas the Superliv herbal formula had a beneficial effect on the growth performance.

The herbal feed additive had an influence on heart development; liver weight tended to decrease.

Reduced energy concentration in broiler chickens diets increased fat content in breast muscles; also, lower MDA concentration was noted.

Reduced energy concentration had a negative effect on water-holding capacity and meat acidity. Meat had a lighter color and lower redness content. Superliv had an effect on meat acidity measured 15 min post mortem (pH${}_{15})$.

Due to the growth performance results, Superliv may be a valuable fodder additive in broiler chicken nutrition.

## Data Availability

The data sets are available upon request from the corresponding author.
